# Genetic Determinants of Ototoxicity During and After Childhood Cancer Treatment: Protocol for the PanCareLIFE Study

**DOI:** 10.2196/11868

**Published:** 2019-03-19

**Authors:** Eva Clemens, Annelot JM Meijer, Linda Broer, Thorsten Langer, Anne-Lotte LF van der Kooi, André G Uitterlinden, Andrica de Vries, Claudia E Kuehni, Maria L Garrè, Tomas Kepak, Jarmila Kruseova, Jeanette F Winther, Leontien C Kremer, Eline van Dulmen-den Broeder, Wim JE Tissing, Catherine Rechnitzer, Line Kenborg, Henrik Hasle, Desiree Grabow, Ross Parfitt, Harald Binder, Bruce C Carleton, Julianne Byrne, Peter Kaatsch, Antoinette am Zehnhoff-Dinnesen, Oliver Zolk, Marry M van den Heuvel-Eibrink

**Affiliations:** 1 Princess Maxima Center for Pediatric Oncology Utrecht Netherlands; 2 Department of Pediatric Hematology and Oncology Erasmus Medical Center–Sophia Children's Hospital Rotterdam Netherlands; 3 Department of Internal Medicine Erasmus Medical Center Rotterdam Netherlands; 4 Department of Pediatric Oncology University Hospital for Children and Adolescents Luebeck Germany; 5 Department of Obstetrics and Gynecology Erasmus Medical Center–Sophia Children's Hospital Rotterdam Netherlands; 6 Institute of Social and Preventive Medicine University of Bern Bern Switzerland; 7 Department of Paediatric Respiratory Medicine University Children's Hospital University of Bern Bern Switzerland; 8 Department of Neurooncology Institute Giannina Gaslini Genova Italy; 9 Department of Paediatric Oncology University Hospital Brno Masaryk University Brno Czech Republic; 10 St. Anne’s University Hospital Brno–International Clinical Research Center Brno Czech Republic; 11 Department of Pediatric Hemato-Oncology Motol University Hospital Prague Prague Czech Republic; 12 Danish Cancer Society Research Center Copenhagen Denmark; 13 Department of Clinical Medicine Faculty of Health Aarhus University Aarhus Denmark; 14 Department of Pediatric Oncology Academic Medical Center Amsterdam Amsterdam Netherlands; 15 Department of Pediatric Hematology and Oncology VU Medical Center Amsterdam Netherlands; 16 Department of Pediatric Oncology University Medical Center Groningen University of Groningen Groningen Netherlands; 17 Department of Pediatrics and Adolescent Medicine Copenhagen University Hospital Rigshospitalet Copenhagen Denmark; 18 Department of Pediatrics Aarhus University Hospital Aarhus Denmark; 19 German Childhood Cancer Registry, Institute of Medical Biostatistics Epidemiology and Informatics University Medical Center of the Johannes Gutenberg University Mainz Mainz Germany; 20 Department of Phoniatrics and Pedaudiology University of Münster Muenster Germany; 21 Institute of Medical Biometry and Statistics Faculty of Medicine and Medical Center University of Freiburg Freibug Germany; 22 Division of Translational Therapeutics, Department of Pediatrics British Columbia Children’s Hospital Research Institute University of British Columbia Vancouver, BC Canada; 23 Boyne Research Institute Drogheda Ireland; 24 Institute of Pharmacology of Natural Products and Clinical Pharmacology Ulm University Medical Center Ulm Germany

**Keywords:** ototoxicity, hearing loss, childhood cancer survivors, cisplatin, genetics, GWAS, candidate genes, polymorphisms

## Abstract

**Background:**

Survival rates after childhood cancer now reach nearly 80% in developed countries. However, treatments that lead to survival and cure can cause serious adverse effects with lifelong negative impacts on survivor quality of life. Hearing impairment is a common adverse effect in children treated with cisplatin-based chemotherapy or cranial radiotherapy. Ototoxicity can extend from high-tone hearing impairment to involvement of speech frequencies. Hearing impairment can impede speech and language and neurocognitive development. Although treatment-related risk factors for hearing loss following childhood cancer treatment have been identified, the individual variability in toxicity of adverse effects after similar treatment between childhood cancer patients suggests a role for genetic susceptibility. Currently, 12 candidate gene approach studies have been performed to identify polymorphisms predisposing to platinum-induced ototoxicity in children being treated for cancer. However, results were inconsistent and most studies were underpowered and/or lacked replication.

**Objective:**

We describe the design of the PanCareLIFE consortium’s work packages that address the genetic susceptibility of platinum-induced ototoxicity.

**Methods:**

As a part of the PanCareLIFE study within the framework of the PanCare consortium, we addressed genetic susceptibility of treatment-induced ototoxicity during and after childhood cancer treatment in a large European cohort by a candidate gene approach and a genome-wide association screening.

**Results:**

This study included 1124 survivors treated with cisplatin, carboplatin, or cranial radiotherapy for childhood cancer, resulting in the largest clinical European cohort assembled for this late effect to date. Within this large cohort we defined a group of 598 cisplatin-treated childhood cancer patients not confounded by cranial radiotherapy. The PanCareLIFE initiative provided, for the first time, a unique opportunity to confirm already identified determinants for hearing impairment during childhood cancer using a candidate gene approach and set up the first international genome-wide association study of cisplatin-induced direct ototoxicity in childhood cancer patients to identify novel allelic variants. Results will be validated in an independent replication cohort. Patient recruitment started in January 2015 and final inclusion was October 2017. We are currently performing the analyses and the first results are expected by the end of 2019 or the beginning of 2020.

**Conclusions:**

Genetic factors identified as part of this pan-European project, PanCareLIFE, may contribute to future risk prediction models that can be incorporated in future clinical trials of platinum-based therapies for cancer and may help with the development of prevention strategies.

**International Registered Report Identifier (IRRID):**

DERR1-10.2196/11868

## Introduction

Survival outcomes after childhood cancer have improved considerably over the last decades, now reaching approximately 80%. This marked increase is a result of advanced diagnostic and treatment procedures, improved stratification options, and optimized supportive care [1]. Nevertheless, more than 25% of all childhood cancer survivors (CCS) are affected with severe or life-threatening long-term side effects of treatment (eg, heart failure, secondary malignant neoplasms, and cognitive dysfunction), and approximately 75% of all CCS experience at least one long-term side effect [2,3]. Ototoxicity is a side effect of childhood cancer treatment and is defined by damage to the cochlea resulting in hearing loss, tinnitus, and/or vertigo [4].

Hearing loss is frequently encountered in childhood cancer patients and survivors treated with platinum derivatives such as cisplatin and carboplatin [5-9]. Studies have shown that 45% to 60% of CCS treated with cisplatin develop irreversible hearing loss and almost half of them may require hearing aids [10,11]. Platinum-induced hearing loss usually starts in the high frequencies but can eventually affect the lower frequencies, including speech frequencies [12].

Even though hearing loss is not a life-threatening disorder, it is a serious adverse effect of treatment, especially in children at ages before and during language acquisition. It can cause distress, anxiety, and depression leading to problems with speech development, neurocognitive functioning, school performance, and social life [6,9,12-14]. Hence, hearing loss can have a large negative and lifelong impact on quality of life [15]. Currently, novel therapeutics such as sodium thiosulfate have proven to be otoprotective, yet they cannot be applied in clinical practice since these novel therapeutics can reduce the efficacy of anticancer treatment [16].

Apart from platinum compounds, several other risk factors for hearing loss during and after childhood cancer therapy have been identified. These include a high platinum dose, renal dysfunction, young age at diagnosis, concomitant use of other potentially ototoxic drugs, and cranial irradiation. However, in total these factors only partially explain the interindividual variability in ototoxic responses to platinum [17]. This suggests that genetic susceptibility may contribute to the occurrence of hearing loss in CCS. Although several genetic association studies have been performed so far, their results are uncertain due to study design, selection of particular candidate genes, failure of independent replications, and/or the small sample size, which limits statistical power. In addition, some studies were heterogeneous with respect to ethnicity and/or the nongenetic risk profile, particularly the inclusion of cranial irradiated cases and the types of platinum compounds [6,18,19]. All but one of the previous studies in pediatric cancer survivors focused on genetic associations within prespecified genes of interest (ie, candidate gene approach), yet it is unclear whether previous studies indeed have considered the most relevant candidate genes.

Prerequisites for a satisfactory approach to identification of genetic determinants of platinum-induced hearing loss would be adequate numbers of research subjects and well-documented clinical and treatment data. The multinational PanCareLIFE (PCL) study provides a unique opportunity to investigate preexisting and novel genetic markers for treatment-related hearing loss in CCS [20]. PCL is funded by the European Union’s Seventh Framework Programme from 2013-2018 and originated from the PanCare network [21]. Investigators from 10 European countries collected data from over 12,000 CCS in order to investigate the determinants of long-term health in this population. PCL addresses three main outcomes: ototoxicity, fertility impairment, and quality of life.

This study is part of the PCL project and addresses the genetic susceptibility of platinum-induced hearing loss. Specifically, the aims were to identify clinical and genetic risk markers in a large cohort of CCS and identify additional genetic risk markers of hearing loss by genome-wide association screening (GWAS) in a carefully characterized subgroup.

## Methods

In total, 8 work packages (WPs) are included in the PanCareLIFE study, of which 5 are scientific WPs. In this study, WP5 and WP4b will be addressed.

### Study Population and Inclusion Criteria

**Figure 1:**
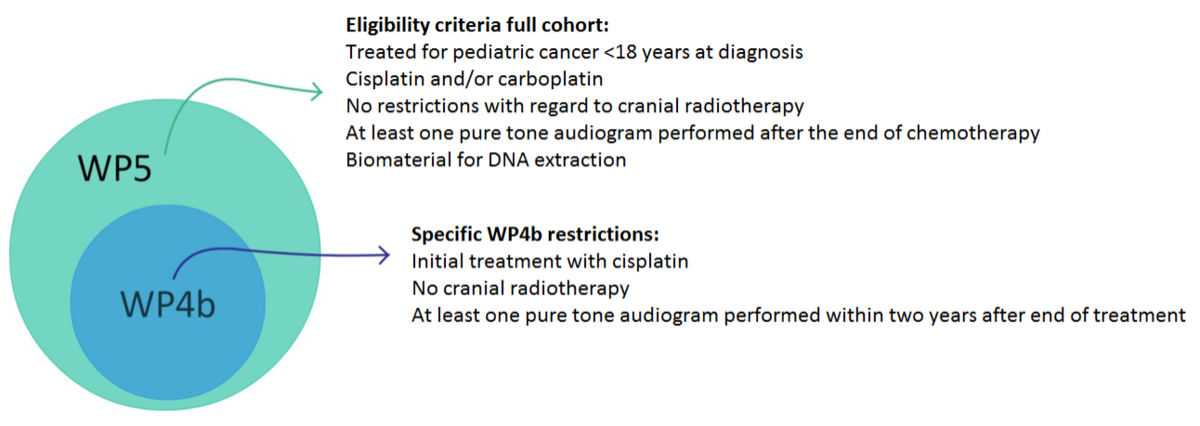
Description of WP4b and WP5 study cohorts.

WP5 aimed to identify clinical and genetic risk markers in a large cohort of CCS. For eligibility in the PCL WP5 genetic study, the following inclusion criteria were applied: (1) patients were younger than age 18 years at cancer diagnosis; (2) patients had been treated for cancer with cisplatin, carboplatin, or both; (3) patients were off therapy and had at least one pure tone audiometric evaluation available after the end of chemotherapy; and (4) patients had provided biomaterial (saliva or blood) for DNA extraction (Figure 1). Subjects were excluded from the study if they had permanent hearing loss identified before the start of cancer therapy.

WP4b used a subset of research subjects from WP5 and aimed to identify additional genetic risk markers of hearing loss by GWAS with better control of nongenetic risk factors. An additional set of inclusion criteria was imposed to reduce potentially confounding factors and focus on the influence of cisplatin. These were (1) cancer treatment included upfront cisplatin, either cisplatin as the single platinum drug throughout the entire course of treatment or change from cisplatin to carboplatin during treatment, and (2) no radiotherapy administration to the brain or inner ear (Figure 1). Research subjects whose initial treatment included carboplatin were excluded.

### Ethics Approval and Consent to Participate

The PanCareLIFE study has been approved by the local ethics committees: Kantonale Ethikkommission Bern, 362/2015; Comitate Etico Regionale, 507REG2014; Ethical Committee University Hospital Brno, June 11, 2016; Ethics Committee Fakultni Nemocnice v Motole, Prague; De Videnskabsetiske Komiteer Region Hovedstaden, H-1-2014-125; Ethikkommission Medizinische Universität Graz, 27-015 ex 14/15; Ethikkommission der Universität Ulm, 160/17; Ethikkommission der Universität zu Lübeck, 14/181; Ethik-Kommission der Ärztekammer Westfalen-Lippe und der Westfälischen Wilhelms-Universität Münster, 2014-619; Medische Ethische Toetsings Commissie Erasmus MC; Medisch Ethische Toetsingscommissie, 2015_202. Informed consent was obtained from the patient or their the legal representatives.

### Data Collection, Storage, and Anonymization

CCS were recruited through an institutional network from several countries in Europe. The participating institutions are referred to as data providers. Each data provider collected retrospective demographic, diagnostic, treatment, and audiometric data from their medical record files and registries. Diagnostic data included the *International Classification of Disease* –coded diagnosis and date of diagnosis (for each tumor). Treatment-related data included information on platinum treatment (eg, platinum compound, dose per cycle, cumulative dose, date of start and stop treatment, and infusion duration) and potentially ototoxic comedication (eg, amikacin, gentamycin, tobramycin, furosemide, vincristine, vancomycin). The data were stripped of all identifiers and assigned a unique PCL-ID number, rendering the data pseudonymous for the investigators of this study. Data providers sent their data to the PCL data center in Mainz (German Childhood Cancer Registry, University Medical Center Mainz, Germany), which also collected and archived the genetic, clinical, and audiological data from the lab and the audiometry center.

### Outcome

The main outcome of this study was hearing function following platinum treatment in pediatric cancer survivors. Diagnosis of hearing loss was based on pure tone audiometry performed at frequencies of 250, 500, 1000, 2000, 4000, 6000, and 8000 Hz [22]. Data providers sent the pseudonymized original audiograms to the audiometry center (Department of Phoniatry and Pediatric Audiology, University Hospital Muenster, Germany) for standardized review. The audiogram assessors were blinded to patient characteristics including their treatment, such as platinum compound, platinum dose, or cranial irradiation. The assessors graded the severity of hearing loss using the Muenster criteria [23,24]. The Muenster criteria considers minimal hearing loss (Muenster grade 1: >10 to ≤20 dB) and allows the detection of early post-cisplatin hearing loss. Clinically relevant hearing loss was defined as Muenster grade ≥2b [24]. The International Society of Pediatric Oncology (SIOP) Boston criteria [25,26] was used as an independent, secondary grading. Clinically relevant hearing loss was defined as SIOP grade ≥2 (Table 1).

**Table 1:** Applied ototoxicity criteria.

Grade	Muenster criteria	SIOP^a^ Boston criteria
0	≤10 dB HL^b^ at all frequencies	≤20 dB HL at all frequencies
1	>10 to ≤20 dB HL at one or more frequencies or tinnitus	>20 dB HL above 4 kHz
2	>20 dB HL at 4 kHz and above	>20 dB HL at 4 kHz and above
	2a: >20 to ≤40 dB	
	2b: >40 to ≤60 dB	
	2c: >60 dB	
3	>20 dB HL at <4 kHz	>20 dB HL at 2 kHz and above
	3a: >20 to ≤40 dB	
	3b: >40 to ≤60 dB	
	3c: >60 dB	
4	≥80 dB at <4 kHz	>40 dB HL at 2 kHz and above

^a^SIOP: International Society of Pediatric Oncology.

^b^HL: hearing loss.

### DNA Collection and Genotyping

Detailed methods for DNA collection and genotyping are described elsewhere [27]. For the first aim of the genotype study, a candidate gene approach was applied to validate 10 previously identified single nucleotide polymorphisms (SNPs) associated with hearing loss in childhood cancer patients and survivors [19,28-40]: *ACYP2*, *LRP2*, *NFE2L2*, *OTOS*, *TPMT*, *SOD2*, *SLC22A2*, *GSTP1*, *ABCC3*, and *SLC16A5* [30,32,35,38,40-43]. In WP4b, array-based genotyping data was used. Thereafter, these data were merged with data from TaqMan PCR generated in WP5. Next, novel SNPs that were independently associated with treatment-related hearing loss in childhood cancer patients were explored within WP4b by GWAS. For maximum standardization, array genotyping in the PCL consortium was conducted by one partner (genetic laboratory of the Department of Internal Medicine in the Erasmus Medical Center, Rotterdam, the Netherlands) [44]. The Infinium Global Screening Array (Illumina, Inc), which contains >770,000 SNPs, was used [45].

### Quality Control and Imputations

A stringent quality control protocol was applied where multiple filters were used to ensure the quality of the genetic data prior to either imputations or analysis. The quality control procedure is described elsewhere [27]. To remove poorly genotyped SNPs and individuals from the data, a call rate of 97.5% was applied. In addition, a Hardy-Weinberg equilibrium test (*P*<1*10^–7^) was assessed to identify potential genotyping errors. Samples with gender mismatches, familial relationships, and extreme heterozygosity were removed to ensure sample quality. After the quality control, principal components were calculated in order to adjust for population heterogeneity and technical confounders in all subsequent analyses [46]. Imputations were performed using the Michigan Imputation Server with default settings [47]. The reference panel chosen for imputations was the Haplotype Reference Consortium (HRC r1.1) [48]. This panel has also been used in large-scale population-based studies such as the Rotterdam Study [49] and Generation R [50].

### Statistical Power

To estimate the number of cases required for the GWAS analyses, a sample size calculation was performed. Assuming a risk allele frequency of 0.2, a case to control ratio of 1:1, and a *P* value threshold of *P*<5*10^–8^ for the GWAS analysis, a cohort of 574 patients was considered sufficient to detect an odds ratio of at least 2.8 with a statistical power of 80% in the design of the study.

### Genetic Susceptibility Analysis

For both candidate gene and GWAS analyses, genetic profiles from children who were treated with cisplatin and have hearing impairment were compared to those of children treated with cisplatin who did not develop hearing impairment. Relationships of categorical data were compared using the chi-square and Fisher exact tests. Comparison of distribution between groups with continuous data was tested with the Mann-Whitney and Kruskal-Wallis tests. Standard logistic regression models adjusting for age at diagnosis, gender, total cumulative cisplatin dose, and principal components were employed to calculate odds ratios with 95% confidence intervals in order to assess the risk of hearing loss. Principal component analysis, a common tool that has been widely used for the combined analysis of correlated phenotypes in genetic linkage and association studies, was used to correct for population stratification by modeling ancestry differences between cases and controls. Bonferroni correction was used in the candidate gene analysis to adjust for multiple testing. In the GWAS, a suggestive significance threshold of *P*<1*10^–6^ was used to identify relevant SNPs that could be important but did not reach genome-wide significance (*P*<5*10^–8^). All statistical analyses were performed by investigators of WP4b and WP5 in close collaboration with the Biostatistical Support Group of UMC Mainz and Ulm.

For both the WP4b candidate gene approach and WP4b GWAS, replication analysis is planned within an independent Canadian cohort from the Canadian Pharmacogenomics Network for Drug Safety (CPNDS).

## Results

**Figure 2:**
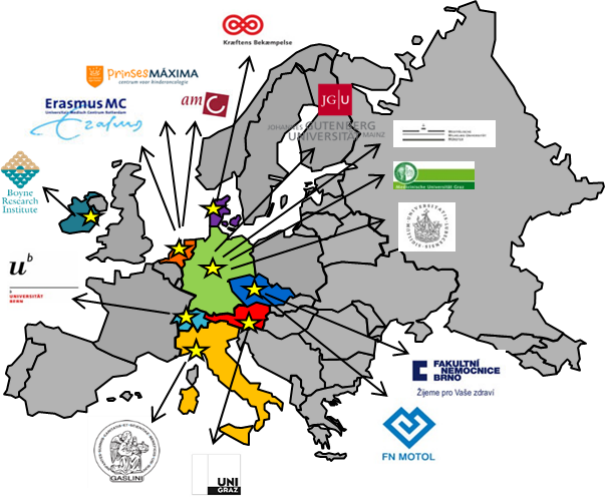
Data providers participating in WP4b and WP5 of PanCareLIFE: University of Bern, Instituto Giannina Gaslini, University Hospital Brno, Motol Teaching Hospital Prague, Kraeftens Bekaempelse, University Graz, Osteosarcoma clinical trial, University Hospital Muenster, Euramos clinical trial, University Lübeck, Academical Medical Center Amsterdam, Erasmus Medical Center Rotterdam, University Medical Center Groningen and Princess Máxima Center for Pediatric Oncology Utrecht. PanCareLIFE study management: Boyne Research Institute Drogheda and German Childhood Cancer Registry Mainz.

Study participants were recruited through a network of 14 institutions from 7 countries: Switzerland, Italy, Czech Republic, Denmark, Germany, Austria, and the Netherlands. The data providers and number of patients per data provider are shown in Table 2; data providers and locations are depicted on a map in Figure 2. WP5 ultimately enrolled a total of 1124 patients. Compared to WP5, WP4b investigated a more restricted study population of 598 patients. Germline DNA, extracted from EDTA blood or saliva samples, was used for genotype studies. To reduce patient discomfort and boost study enrollment, saliva was allowed as an alternative to blood. In total, the data providers collected a similar number of blood and saliva samples. Biosamples were stored and processed at the University Medical Center Ulm, Germany, and at the Erasmus Medical Center Rotterdam, the Netherlands, until analysis. Blood samples were stored at –20°C or lower; saliva samples were stored at room temperature. Germline DNA was extracted using the salting-out method and served as a template for TaqMan polymerase chain reaction (PCR; WP5) and array-based genotyping (WP4b).

Patient recruitment started in January 2015 and final inclusion was October 2017. We are currently performing the analyses and the first results are expected by the end of 2019 or the beginning of 2020.

**Table 2:** Data providers included in the genetics study.

Data provider	Country	Patients enrolled in WP5^a^	Patients enrolled in WP4b^b^
University of Bern, Bern	Switzerland	153	73
Istituto Giannina Gaslini, Genova	Italy	8	8
University Hospital Brno, Brno	Czech Republic	173	54
Motol Teaching Hospital Prague, Prague	Czech Republic	86	41
Kraeftens Bekaempelse, Copenhagen	Denmark	94	35
Osteosarcoma clinical trial	Germany	124	107
University Hospital Muenster, Muenster	Germany	297	111
Euramos clinical trial	Germany	36	34
University Lübeck, Lübeck	Germany	30	12
University Graz, Graz	Austria	29	10
Academical Medical Center Amsterdam, Amsterdam	The Netherlands	17	22
Erasmus Medical Center, Rotterdam	The Netherlands	32	32
University Medical Center Groningen, Groningen	The Netherlands	18	18
Princess Máxima Center for Pediatric Oncology, Utrecht	The Netherlands	27	27

^a^WP5: work package 5.

^b^WP4b: work package 4b.

## Discussion

### Principal Findings

This paper describes the design of the PCL ototoxicity genetics study aiming to identify clinical and genetic risk markers in a large and heterogeneous cohort of CCS and identify additional genetic risk markers of hearing loss by GWAS with better control of nongenetic risk factors in a more homogenous subcohort of CCS. Data were collected from 1124 CCS from 7 different European countries. Some of the previously identified genetic variants for hearing loss were validated by a candidate gene approach. In addition, the first international GWAS of cisplatin-induced hearing loss sets out to identify novel allelic variants in the largest European cohort assembled for such a genome-wide pharmacogenetics association study so far.

For this study, a subcohort was recruited consisting of patients who were treated with cisplatin and did not receive cranial irradiation, a well-known independent risk factor for sensorineural hearing loss. From 14% to 27% of children who received radiotherapy without ototoxic chemotherapy suffered from high-frequency hearing loss [51,52]. This risk of hearing loss increases in patients who require platinum-based chemotherapy combined with radiation. Whether the same genetic markers are associated with platinum- and radiation-induced hearing loss is unknown. To limit contamination by the presence of confounding factors such as cranial irradiation, a more homogenous subcohort of patients was selected for WP4b.

Appropriately sized cohorts are required to identify genetic determinants of platinum-induced hearing loss. The many associations that are tested in a GWAS require a very low significance threshold to prevent an inflated genome-wide type I error. This reduces the probability of identifying SNPs with small effect size, unless sample sizes are large enough to achieve sufficient power to identify such SNPs. The large combined cohort within the PCL consortium is expected to provide adequate statistical power.

Many classification systems of drug-induced hearing loss have been developed—Brock grading system [53], American Speech-Language-Hearing Association (ASHA) criteria [54], Chang classification [55], Muenster classification [24], and the SIOP Boston Ototoxicity Grading Scale [26]—yet an international standard for ototoxicity reporting is still lacking. Choice of the classification system and definitions of hearing loss may have an impact on the frequency of occurrence in childhood cancer survivors [56], as shown in a recent study that investigated the influence of several classification methods in a large prospective cohort of platinum-treated children and adolescents. Estimates of the overall occurrence of hearing loss (40% to 56%) and severe hearing loss (7% to 22%) cover a wide range [57]. Compared to other methods, Muenster grade 1 is considered a strong predictor for the need of hearing support in CCS, with reported sensitivity and specificity levels of 67% and 87%, respectively [58]. In addition, the SIOP Boston scale might be superior to determine hearing loss compared to the ASHA, Brock, and Common Terminology Criteria for Adverse Events (CTCAE) methods, based on the high number of evaluable assessments, sensitivity, and earliest time to detect hearing loss [57]. In order to strengthen our study, both the Muenster classification and SIOP Boston scale were used for a valid interpretation of the severity of platinum-induced hearing loss in this cohort.

The availability of a large European set of clinical, audiometric, and genetic data provides the PCL consortium excellent opportunities for further collaboration, including replication studies in independent transatlantic cohorts or meta-analyses. In order to validate findings from the initial discovery cohort, it is standard practice to include an independent replication cohort. A collaboration with the CPNDS for replication of results of this study has been initiated. The patients enrolled in our replication cohort were recruited from 11 hospitals and health care centers in Canada. Hearing loss in the CPNDS cohort was originally graded according to the CTCAE [29]. Applying a standardized definition for hearing loss facilitates a combined analysis of the CPNDS and PCL data. For that purpose, end point harmonization was pursued by reevaluating all audiograms (EC) of the CPNDS cohort according to Muenster and SIOP criteria. Additional replication cohorts could be needed for future international collaborations.

### Limitations

The PanCareLIFE ototoxicity studies have some limitations. As a result of missing or unclassifiable audiograms, some patients cannot be included due to a missing phenotype. Because many of the patients with missing audiograms might have good hearing function, they might therefore no longer be followed up for audiometric testing, As a consequence, the risk of ototoxicity based on the results of this study might be an overestimation of the true risk. Currently, the International Late Effects of Childhood Cancer Guideline Harmonization Group is developing recommendations for audiological monitoring in CCS. The guideline unifies existing recommendations and provides optimum follow-up practices, which is important for consensus on the frequency and timing of audiological evaluations after childhood cancer [59].

### Conclusions

In summary, our paper described the design of a genetic susceptibility study that addresses an important late effect of cancer therapy (ie, platinum-induced hearing loss in survivors of cancer diagnosed and treated during childhood). Identification of genetic risk factors may assist in the development of more accurate prediction models that can be incorporated in future clinical trials of platinum-based therapies for cancer. Increased knowledge of nongenetic and genetic risk factors of cisplatin-induced hearing loss may contribute to the development of preventive methods to improve quality of life in CCS.

## Abbreviations

ASHA: American Speech-Language-Hearing Association
CCS: childhood cancer survivors
CPNDS: Canadian Pharmacogenomics Network for Drug Safety
CTCAE: US National Cancer Institute Common Terminology Criteria for Adverse Events
GWAS: genome-wide association screening
PCR: polymerase chain reaction
PCL: PanCareLIFE
SIOP: International Society of Pediatric Oncology
SNP: single nucleotide polymorphism
WP: work package
WP4b: Work Package 4b
WP5: Work Package 5
